# Identification of phlebotomine sand flies using one MALDI-TOF MS reference database and two mass spectrometer systems

**DOI:** 10.1186/s13071-015-0878-2

**Published:** 2015-05-10

**Authors:** Alexander Mathis, Jérôme Depaquit, Vit Dvořák, Holly Tuten, Anne-Laure Bañuls, Petr Halada, Sonia Zapata, Véronique Lehrter, Kristýna Hlavačková, Jorian Prudhomme, Petr Volf, Denis Sereno, Christian Kaufmann, Valentin Pflüger, Francis Schaffner

**Affiliations:** Swiss National Centre for Vector Entomology, Institute of Parasitology, University of Zürich, Winterthurerstrasse 266a, 8057 Zürich, Switzerland; Université de Reims Champagne-Ardenne, ANSES, EA4688 - USC “transmission vectorielle et épidémiosurveillance de maladies parasitaires (VECPAR)”, SFR Cap Santé, 51, rue Cognacq-Jay, 51096 Reims, France; Department of Parasitology, Faculty of Sciences, Charles University in Prague, Vinicna 7, 128 44 Prague 2, Czech Republic; MIVEGEC, UMR CNRS-IRD-Université de Montpellier Centre IRD, 911 Avenue Agropolis, BP 64501, , Cedex 34394 Montpellier, France; Laboratory of Molecular Structure Characterization, Institute of Microbiology, The Czech Academy of Sciences, Videnska 1083, 142 20 Prague 4, Czech Republic; Instituto de Microbiología, Universidad San Francisco de Quito, Diego de Robles S/N, Quito, Ecuador; Mabritec SA, Lörracherstrasse 50, 4125 Riehen, Switzerland; Avia-GIS, Risschotlei 33, 2980 Zoersel, Belgium; Current address: NSF Center for Integrated Pest Management, 1730 Varsity Drive, Suite 110, Venture IV Building, Raleigh, North Carolina 27606 USA

**Keywords:** Arthropod identification, Bruker, Centralized reference database, Cross reference, Phlebotominae, Protein Profiling, Shimadzu, Spectra processing, Validation

## Abstract

**Background:**

Rapid, accurate and high-throughput identification of vector arthropods is of paramount importance in surveillance programmes that are becoming more common due to the changing geographic occurrence and extent of many arthropod-borne diseases. Protein profiling by MALDI-TOF mass spectrometry fulfils these requirements for identification, and reference databases have recently been established for several vector taxa, mostly with specimens from laboratory colonies.

**Methods:**

We established and validated a reference database containing 20 phlebotomine sand fly (Diptera: Psychodidae, Phlebotominae) species by using specimens from colonies or field-collections that had been stored for various periods of time.

**Results:**

Identical biomarker mass patterns (‘superspectra’) were obtained with colony- or field-derived specimens of the same species. In the validation study, high quality spectra (i.e. more than 30 evaluable masses) were obtained with all fresh insects from colonies, and with 55/59 insects deep-frozen (liquid nitrogen/-80 °C) for up to 25 years. In contrast, only 36/52 specimens stored in ethanol could be identified. This resulted in an overall sensitivity of 87 % (140/161); specificity was 100 %. Duration of storage impaired data counts in the high mass range, and thus cluster analyses of closely related specimens might reflect their storage conditions rather than phenotypic distinctness. A major drawback of MALDI-TOF MS is the restricted availability of in-house databases and the fact that mass spectrometers from 2 companies (Bruker, Shimadzu) are widely being used. We have analysed fingerprints of phlebotomine sand flies obtained by automatic routine procedure on a Bruker instrument by using our database and the software established on a Shimadzu system. The sensitivity with 312 specimens from 8 sand fly species from laboratory colonies when evaluating only high quality spectra was 98.3 %; the specificity was 100 %. The corresponding diagnostic values with 55 field-collected specimens from 4 species were 94.7 % and 97.4 %, respectively.

**Conclusions:**

A centralized high-quality database (created by expert taxonomists and experienced users of mass spectrometers) that is easily amenable to customer-oriented identification services is a highly desirable resource. As shown in the present work, spectra obtained from different specimens with different instruments can be analysed using a centralized database, which should be available in the near future via an online platform in a cost-efficient manner.

## Background

The geographic occurrence and extent of arthropod-borne diseases is changing, due to globalisation and environmental alterations. Most spectacular is the emergence of invasive species, e.g. *Aedes* mosquitoes [1, 2], with associated new disease threats [3]. Other arthropod vectors such as ticks (Ixodida: Ixodidae) [4, 5], biting midges (Diptera: Ceratopogonidae) [6] and phlebotomine sand flies (Diptera: Psychodidae) [7–9] are gradually expanding their distribution ranges in Europe. Because these changes in vector distribution are associated with changes in the risk of exposure to the pathogens they transmit, monitoring the presence and abundances of arthropod vectors is of growing importance in many regions. Guidelines for surveillance and options for control have so far only been developed for mosquitoes, both native and invasive species [2, 10, 11]. Regularly updated distribution maps for Europe are provided by ECDC (www.ecdc.europa.eu) for a number of vectors (eight sand fly species, five *Aedes* mosquito species, four tick species).

Undoubtedly, reliable, rapid and cost-effective identification of vector arthropods is a key element of surveillance programmes. Morphological identification requires at least some degree of proficiency and can be time-consuming, e.g. requiring slide preparations and in-depth analysis of characteristics such as the morphology of pharynx and genitalia in the case of sand flies [12]. However, morphological identification can be difficult or impossible in many instances (e.g. due to specimens of sibling or cryptic species; damaged or incomplete specimens; life stages with few morphological features like eggs or larvae; poor preservation methods or damage during shipment). In the case of sand flies, which are the focus of this paper, morphological identification is sometimes hampered by the lack of comprehensive keys, minute species-distinctive characters in some subgenera and the existence of phenotypic plasticity among populations [13, 14]. PCR-based approaches are established in different formats for a number of phlebotomine species (compiled by [15, 16]), but their validity may be impaired due to genetic variability at the target locus [17, 18]. Further, these approaches are generally considered expensive and time-consuming.

As an alternative, protein profiling by matrix-assisted laser desorption/ionization time of flight mass spectrometry (MALDI-TOF MS), which is routinely used in clinical diagnostics of bacteria and fungi [19, 20] with high interlaboratory reproducibility [21], has recently been applied for the identification of a number of arthropods, including adult stages of vector taxa [15, 22–33]. In addition, protein profiles have been determined for larvae of holometabolous *Culicoides* biting midges and Culicidae [34, 35], and for eggs of nine aedine mosquito species [36]. MALDI-TOF MS was capable of identifying sister taxa and cryptic species [26, 28], and the method proved reliable for large scale species identification of *Culicoides* biting midges (correct identification of 98.9 % of 1,200 field-collected specimens) as well as in the surveillance of invasive mosquito species (identification of eggs from ovitraps; [28]).

This paper elaborates on the suitability of MALDI-TOF MS to identify adult phlebotomine sand flies, the biological vectors of a number of important protozoan and viral pathogens of medical or veterinary importance [7, 37, 38], extending the method from identifying specimens from laboratory colonies [15] to identifying specimens collected in the field and stored for a prolonged period of time. In addition, we evaluated whether raw mass spectra obtained with the instrument of one company (Ultraflex III, Bruker, Germany) allow species identification by using the software and the reference database established on an instrument of another company (Axima Confidence, Shimadzu, Japan), whereby the two most widespread instrument series were involved. The ability to cross-reference specimens with non-parent databases would strongly enhance the value and availability of in-house reference databases established at different institutions.

## Methods

### Sand fly collection and morphological identification

Mass spectra were determined from sand fly specimens of 20 species obtained from field collections (9 species), colonies (7) or both (4) (see Table 1 for origin, year of collection). Sand flies were captured using CDC miniature light traps (John W. Hock Co. FL, USA) or sticky papers. Specimens were killed using carbon dioxide or liquid nitrogen and stored as described in Table 1. For morphological identification, head and genitalia of each specimen were processed and mounted on slides following traditional procedures [39] and the species determined using standard keys [40–42]. The taxonomic abbreviations used are as described [43].

**Table 1:** Features of the phlebotomine sand flies investigated in the experimental part of the study

Species (morphological ID)	Origin (field; colony)	Collection year; storage until analyses in 2014	No. used for calculating reference database	No. used in validation study
*Lutzomyia* (*Lutzomyia*) *longipalpis* ^1^	Brazil (colony^3^)	2014; 70 % EtOH for few days	5	5
*Nyssomyia trapidoi* ^1^	Ecuador (field)	2013; 96 % EtOH	5	5
*Phlebotomus* (*Adlerius*) *arabicus* ^1^	Israel (colony^3^)	2014; 70 % EtOH for few days	5	5
*Phlebotomus* (*Euphlebotomus*) *argentipes* ^1^	India (colony^3^)	2014; 70 % EtOH for few days	5	5
*Phlebotomu*s (*Larroussius*) *ariasi* ^1^	Southern France (field)	2011; 70 % EtOH, −20 °C	5	7
*Phlebotomus* (*Euphlebotomus*) *barguesae* ^1^	Thailand (field)	2012; 96 % EtOH	5	6
*Phlebotomus* (*Phlebotomus*) *duboscqi*	Senegal (colony)	2002; N_2_ then −80 °C^4^.	0	12
*Phlebotomus* (*Phlebotomus*) *duboscqi* ^1^	Senegal (colony^3^)	2014; 70 % EtOH for few days	5	5
*Phlebotomus* (*Transphlebotomus*) *mascittii* ^1^	France (colony)	2004; N_2_ then −80 °C^4^.	5	8
*Phlebotomus* (*Larroussius*) *neglectus* ^1^	Italy (field)	1991; N_2_ then −80 °C^4^.	5	2
*Phlebotomus* (*Larroussius*) *orientalis* ^1^	Ethiopia (colony^3^)	2014; 70 % EtOH for few days	5	5
*Phlebotomus* (*Phlebotomus*) *papatasi*	Saudia Arabia (colony)	1994; N_2_ then −80 °C^4^.	0	7
*Phlebotomus* (*Phlebotomus*) *papatasi* ^1^	Turkey (colony^3^)	2014; 70 % EtOH for few days	5	5
*Phlebotomus* (*Larroussius*) *perfiliewi transcaucasicus* ^1^	Iran (field)	2010; 70 % EtOH	5	4
*Phlebotomus* (*Larroussius*) *perniciosus*	Southern France (field)	2011; 70 % EtOH, −20 °C	0	6
*Phlebotomus* (*Larroussius*) *perniciosus* ^*2*^	Italy (field)	1991; N_2_ then −80 °C^4^.	0	11
*Phlebotomus* (*Larroussius*) *perniciosus* ^2^	Malta (field)	1989; N_2_ then −80 °C^4^.	0	12
*Phlebotomus* (*Larroussius*) *perniciosus* ^1^	Spain (colony^3^)	2014; 70 % EtOH for few days	5	5
*Phlebotomus* (*Paraphlebotomus*) *sergenti*	Iran (field)	2010; 70 % EtOH		2
*Phlebotomus* (*Paraphlebotomus*) *sergenti* ^1^	Turkey, Israel (colony^3^)	2014; 70 % EtOH for few days	5	5
*Phlebotomus* (*Larroussius*) *tobbi* ^2^	Iran (field)	2010; 70 % EtOH	0	10
*Phlebotomus* (*Larroussius*) *tobbi* ^1^	Turkey (colony^3^)	2014; 70 % EtOH for few days	5	5
*Phlebotomus (Larroussius) tobbi*	Greece (field)	1990; N_2_ then −80 °C^4^	0	2
*Phlebotomus* sp.	Iran (field)	2010; 70 % EtOH	0	1
*Psathyromyia* (*Foratiniella*) *aragaoi* ^1^	Ecuador (field)	2013; 96 % EtOH	5	0
*Psychodopygus panamensis* ^1^	Ecuador (field)	2013; 96 % EtOH	5	2
*Sergentomyia* (*Sergentomyia*) *dentata* ^1^	Israel (field)	2000; N_2_ then −80 °C^4^.	5	7
*Sergentomyia* (*Sergentomyia*) *minuta* ^1^	Southern France (field)	2011; 70 % EtOH, −20 °C	5	7
*Sergentomyia* (*Sergentomyia*) *schwetzi* ^1^	Ethiopia (colony^3^)	2014; 70 % EtOH for few days	5	5
		Total	100	161

^1^Used for calculating reference database

^2^Used for calculating alternative reference database (replacing spectra from colony insects, see also Fig. 2)

^3^Colonies maintained at Department of Parasitology, Charles University, Prague, Czech Republic [15]

^4^-80 °C since 2012

### Generation of MALDI-TOF MS biomarker mass sets, validation study

Thoraxes with wings and legs were manually homogenized, mixed with matrix and spotted on steel target plates as described [26]. Protein mass fingerprints were obtained using a MALDI-TOF Mass Spectrometry Axima™ Confidence machine (Shimadzu-Biotech Corp., Kyoto, Japan) and analysed with SARAMIS™ Premium software (spectral archive and microbial identification system, AnagnosTec, Potsdam-Golm, Germany) as described elsewhere [36]. Biomarker mass patterns, called ‘superspectra’, were calculated using the SARAMIS™ SuperSpectra™ tool with 5 specimens per species (Table 1) with 4 technical replicates each (quadruplicates). For SuperSpectra™ validation, 161 specimens (Table 1) were analysed (also in quadruplicates), and the generated mass fingerprints were imported into SARAMIS™ software for automated identification with SuperSpectra™. The threshold for identification was set at 75 % biomarker matches based on the reference data set, according to the SARAMIS™ user guideline. Spectra containing less than 30 data counts were considered low quality. A dendrogram was produced as described [27].

### Analyses of raw data mass spectra obtained on Bruker mass spectrometer with Shimadzu software

Mass fingerprints generated using automatic routine procedures on an Ultraflex III MALDI TOF mass spectrometer (Bruker Daltonics, Bremen, Germany) [15] were analysed in a blinded manner with the SARAMIS™ Premium software. Bruker mass lists were exported as mzXml files, adapted to the SARAMIS™ Premium file format by use of an in-house phyton script and imported for automated identification. Mass lists obtained with insect thoraxes were available for 312 specimens of *Phlebotomus arabicus* (n = 13), *Ph. argentipes* (n = 20), *Ph. duboscqi* (n = 10), *Ph. orientalis* (n = 10), *Ph. papatasi* (n = 84), *Ph. perniciosus* (n = 94), *Ph. sergenti* (n = 22), *Ph. tobbi* (n = 59) (from colonies maintained in Prague and analysed after storage in 70 % EtOH overnight [15]) and from 55 field-collected (Karpathos island, Greece, stored in 70 % EtOH for several weeks) *Ph. neglectus* (n = 44); *Ph. similis* (n = 8); *Ph. alexandri* (n = 1), *Ph. galilaeus* (n = 2) (total 367 specimens).

### Genetic analyses

DNA was isolated from the remains of the abdomens with a kit (Qiamp DNA mini kit, Qiagen, Hildesheim, Germany) according to the manufacturer’s instruction and after mechanical homogenization as previously described [44]. Genetic characterisation by PCR/sequencing was mainly done at the mitochondrial cytochrome b gene (cyt b). The primers CB3_PDRmod (5’-CTC CYC ATA TYC AAC CWG AAT G-3’) and CB_R06mod (TAT CTA ATG KTT TCA AAA CAA TTG C-3’) were modified from described ones [45,46]. Species for which no corresponding cyt b sequence was available in GenBank were characterized at the mitochondrial cytochrome c oxidase subunit I gene (COI) using primers LCO1490/HCO2198 [47] or at the small subunit nuclear ribosomal RNA gene using forward primer F2 [48] and the new reverse primer R2_new (5’-GTC CTA TTC CAT TAT TCC ATG C-3’). Direct sequencing of the amplicons was performed by a private company (Synergene, Schlieren, Switzerland).

## Results

### Reference database

Biomarker mass patterns (‘superspectra’) were calculated for 20 sand fly species, by using 5 insects per species. Specimens from colonies (10 species) and insects collected in the field (10 species) were used (Table 1). Reference mass peaks were in the range of 4000 and, depending on the species, 9800 to 11400 Da. Identification of the field-collected species could be confirmed by PCR/sequencing with 1 specimen per species for 8 of these 10 species. The two remaining species were *Ph. barguesae* and *Psathyromyia aragaoi*. In the case of *Ph. barguesae*, the partial (around 570 bp) sequence of the COI gene obtained from the field-collected specimens best matched with the two GenBank sequences ascribed to this species (acc. nos. FJ348734-5), but differed by 7 %. Another 5 specimens morphologically attributed to this species were then genetically characterized, yielding identical sequences. No single sequence was available in GenBank for *Ps. aragaoi*, and partial cyt b and COI gene sequences have been deposited [GenBank: KP763471, GenBank: KP763472].

Superspectra were calculated for the species *Ph. perniciosus* and *Ph. tobbi* with 5 specimens each from either colonies or the field (Table 1), yielding identical masses.

### Validation

The reference database was validated with 161 specimens from 19 species (Table 1). Eighty-four specimens were field-collected and stored in various media, 50 specimens were fresh from ongoing colonies of 10 species (that were also used to create the reference database), and 27 specimens were stored deep-frozen from earlier colonies. Altogether, 140 insects were correctly identified, including 77 specimens from colonies. Poor quality spectra (less than 30 data counts) were obtained with 20 field-collected specimens, of which 17 yielded no result upon automated identification with SuperSpectra™. The analyses of the other 3 of these 20 low data count specimens produced discrepant identifications (in 2 of the 4 technical replicates, no identification in the other two) as compared to morphological and genetic identification. Further, 2 insects probably were mislabelled as *Ph. perniciosus*, as both mass spectrometry and DNA sequencing identified them as *Ph. ariasi*, and specimens of this species had been obtained from the same source. Finally, one specimen yielded a novel spectrum but was identified by morphology as *Ph. perfiliewi*, which is included in the database, and as belonging to the *Ph. perfiliewi* complex by DNA analyses.

Thus, overall sensitivity of MALDI-TOF MS was 87 % (140/161); specificity (defined as 100 minus percentage of wrong identifications as compared to morphological identification, all specimens considered that yielded an MS identification) was 97 % (139/144) or 100 % (when consequently omitting low data count specimens and considering the probable mislabelling in 2 cases).

In addition to the specimens with discrepant identification, 22 arbitrarily chosen specimens were genetically analysed, confirming morphological and MALDI-based identification. The specimen with inconclusive morphological identification (Table 1) was among the specimens with low data count but turned out to belong to the *Ph. perfiliewi* complex based on DNA sequence analysis.

**Fig. 1:**
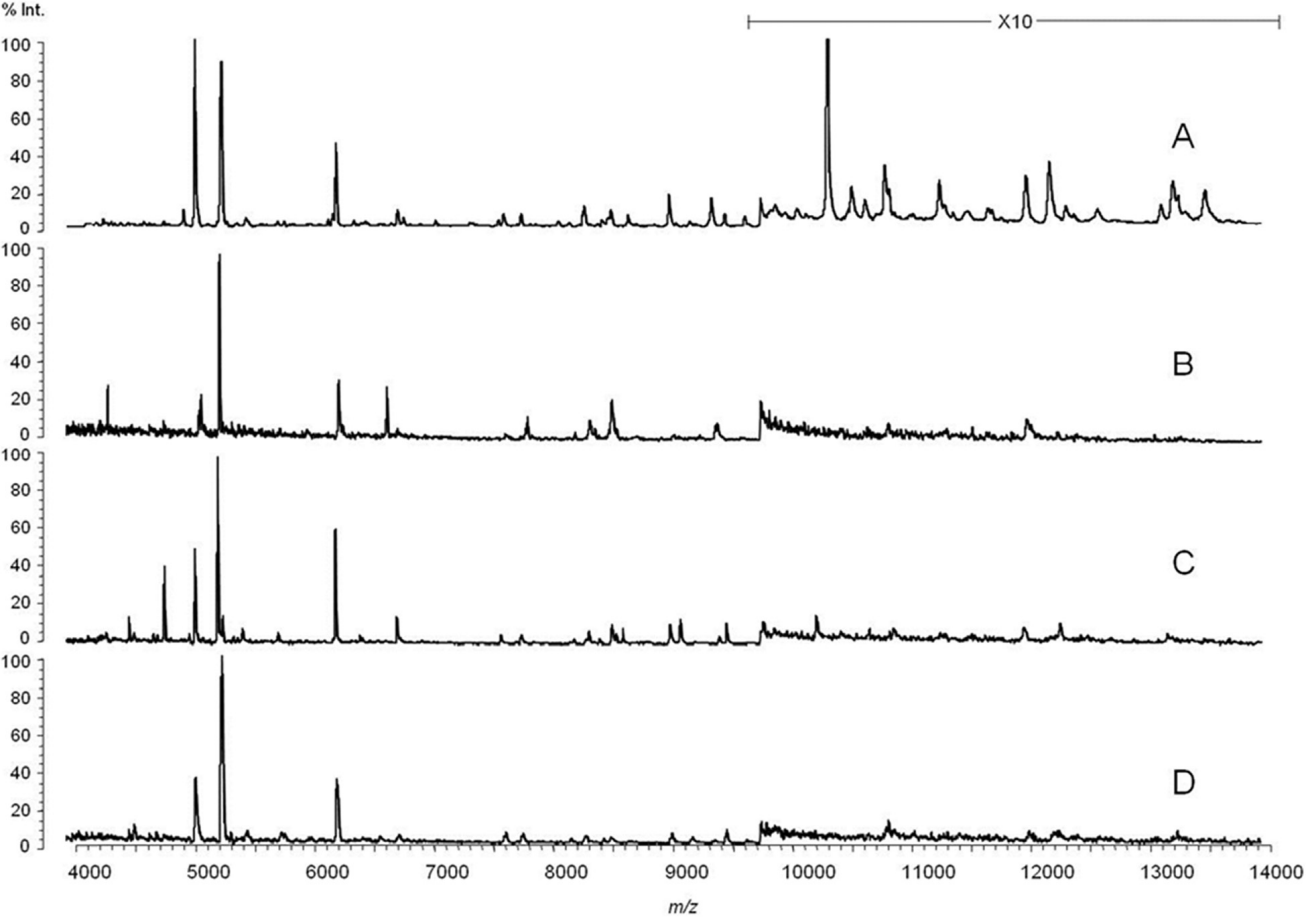
Matrix-assisted laser desorption/ionization time of flight (MALDI-TOF) mass spectra of *Phlebotomus perniciosus*. (**a**) Fresh specimen from colony. (**b**) Field specimen stored in 70 % EtOH, −20 °C for 3 years. (**c**) Field specimen stored in N_2_ then −80 °C since 1991. (**d**) Field specimen stored in N_2_ then −80 °C, since 1989. Note different scale of magnification (10 ×) for the higher mass range (10–20 kDa)

High quality spectra (i.e., equal to or more than 30 data counts) were obtained with all (n = 50) fresh insects from colonies, and with 55/59 insects from colonies or the field and stored deep-frozen (liquid nitrogen/-80 °C) for up to 25 years. In contrast, only 36/52 specimens stored in EtOH (70 or 95 %; room temperature or −20 °C) for only a few years could be identified (Table 1). Duration of storage impaired data counts in the high mass range as exemplified in Fig. 1.

**Fig. 2:**
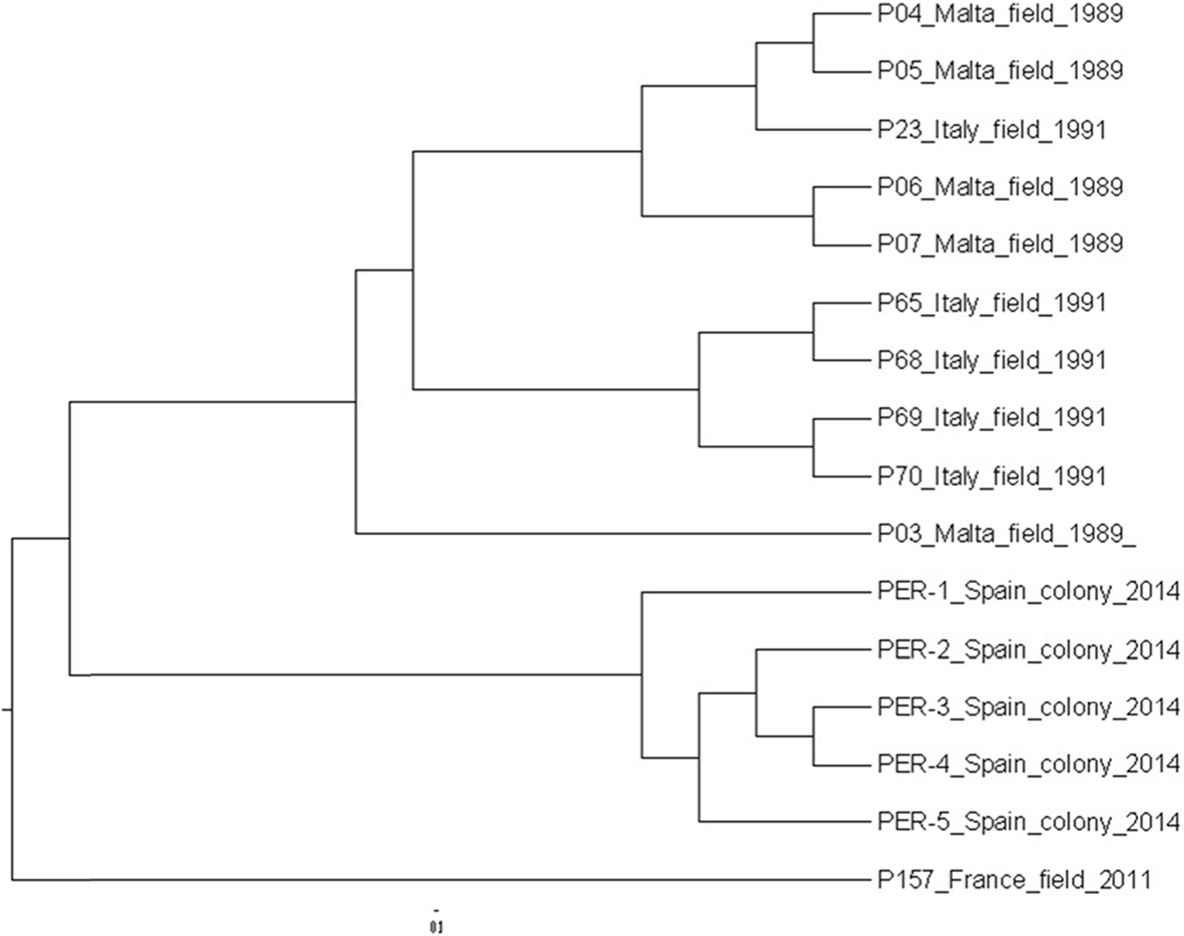
Dendrogram of matrix-assisted laser desorption/ionization time of flight (MALDI-TOF) mass spectra (paired-group dice algorithm) of *Phlebotomus perniciosus* specimens from different groups (colony, geographical origin of field specimens; for conditions and time of storage, see Table 1)

Different batches of sand fly specimens (colony, wild catches from different geographical origins and stored under different conditions and/or for different periods of time) were available for a few species. For example, a dendrogram of *Ph. perniciosus* groups is given in Fig. 2. The spectra from colony insects (originally from Spain) form a distinct cluster, whereas spectra of specimens from two geographical origins (Italy, Malta) and stored under comparable conditions do not. A single spectrum available from a more recently collected *Ph. perniciosus* from France, which was stored differently, is placed on a distinct branch, separated from both the colony- but also the other field-derived spectra.

### Analyses of mass lists obtained on a Bruker instrument with a reference database (SARAMIS™) and reference spectra acquired on a Shimadzu instrument

Raw data from a total of 312 specimens from 8 sand fly species from laboratory colonies were analysed, resulting in the correct identification of 297 individuals. No identification was obtained for 15 specimens, of which 10 had low data count spectra (less than 30). Five spectra with a sufficient number of data counts (30 or more) could not be identified. No misidentification occurred (overall sensitivity 95.2; sensitivity considering only high quality spectra 98.3 %; specificity 100 %). Thirty-six of the 55 field-collected specimens from 4 species were identified in concordance with the morphological identification. The single specimen of *Ph. alexandri* had a high quality spectrum (data count of 57), but yielded no identification as this species was not included in the database. Seventeen spectra contained insufficient data counts and were not identified. One specimen morphologically identified as *Ph. galilaeus* yielded a spectrum identified as *Ph. perniciosus*. Thus, diagnostic parameters with the field-collected specimens differed slightly from laboratory collections (sensitivity considering only high quality spectra 94.7 %; specificity 97.4 %).

## Discussion

### MALDI-TOF MS reference database and validation

A MALDI-TOF MS database was established with 20 phlebotomine species. In selecting our study species, we took into account i) their vectorial role - in order to include many species which are proven vectors of *Leishmania* spp., and ii) their taxonomic position - in order to compare closely related species and species belonging to different genera (Table 1). An evaluation of the database with 161 specimens revealed a robust sensitivity and a very high specificity of this approach. Twenty specimens were not (n = 17) or incorrectly (n = 3) identified due to low spectra quality (data count below 30). Thus, a threshold of 30 data counts, based on our experience with mass spectrometry analyses of Ceratopogonidae biting midges [26, 27], seems a useful criterion to exclude spectra from future analyses.

The major factor impairing mass spectrum quality seems to be the way the insects were stored rather than the duration of storage, though this aspect has not systematically been addressed in the present study. Thus, e.g. 22/23 specimens of *Ph. perniciosus* stored for 25 years in liquid nitrogen/-80 °C could be identified, but only 3/6 insects of the same species stored since 2011 in 70 % EtOH at −20 °C could be identified. This confirms earlier findings that freezing is superior to EtOH as a storage medium, with EtOH at a concentration of 70 % being more suitable than higher concentrations, as experimentally determined with sand flies stored up to 75 days [15]. Thus, this ‘storage constraint’ limits the value of mass spectrometry for the identification of insects, as ethanol is the widely preferred storage medium. However, a high reliability of mass spectrometry (98.9 % good quality spectra) was found with 1,200 field-collected *Culicoides* specimens stored in 70 % EtOH at 4 °C for one year [49]. Most other studies on mass spectrometry as a tool for the identification of arthropods relied on fresh colony-derived specimens. Successful identification by MALDI-TOF MS with field-collected specimens was reported with ticks (stored in liquid nitrogen, [25]), tsetse flies (using air-dried wings, [50]; and mosquitoes (analysis of legs, storage conditions not described, [31]).

As also shown in our study, storage reduces the higher weight masses (Fig. 1). Identification to species level is unaffected by this constraint as reference masses are in the lower mass range. Identification of specimens from different geographic origins (given they were properly stored) was equally reliable by using superspectra derived with either insects fresh from colonies or wild-catches from storage (Table 1). However, higher resolution (e.g., ‘fine typing’ with regard to geographical origin of specimens or lower taxonomic levels) might be critical as the topography of a dendrogram created in cluster analyses could reflect the storage conditions of the analysed specimens rather than phenotypic distinctness (see also Fig. 2). The identification of cryptic insect species (*Anopheles* spp*.*) and even the classification of specimens from different laboratory colonies were possible with a standardized approach (fresh specimens, same feed etc.) [28], but seem improbable with field-collected specimens. Thus, care should be taken not to over-interpret cluster analyses of mass spectra obtained from field-collected and stored specimens.

A novel spectrum was obtained for a specimen that morphologically was identified as a species (*Ph. perfiliewi*) that is included in the database. This species is known to exist as a complex of species [12] which might explain variability in mass spectra. All morphologically identified *Ph. barguesae* specimens had considerable genetic differences at the barcoding locus from corresponding GenBank entries. Genetic variability of this species, which is characterized by a unique morphology of the female spermathecae [51], has been observed among populations from different caves (Depaquit, unpublished). Further studies employing mass spectrometry to different populations of freshly collected specimens and the comparison with results of DNA-based molecular approaches might contribute to elucidate taxonomic relationships. This technique shall be especially considered when dealing with species complexes like *Ph. perfiliewi* or *Ph. major* which are incriminated in transmission of medically important *Leishmania* species and represent a taxonomic challenge.

### Analyses of mass lists obtained on a Bruker instrument with a reference database (SARAMIS™) and reference spectra acquired on a Shimadzu instrument

Comparative studies have shown that the performance of the two systems (Shimadzu, Bruker) for the identification of microorganisms in clinical laboratories is comparable [52, 53]. Exchangeability of data obtained on the two different instruments, however, has to the best of our knowledge not been reported so far. Here, we show that fingerprints of phlebotomine sand flies obtained by automatic routine procedure on a Bruker instrument allow for reliable (high values of diagnostic parameters) determination of species by using the database and the software (SARAMIS™) established on a Shimadzu instrument. The sensitivity of this analysis was high despite slight differences in sample preparation [15] and mass ranges considered (Bruker: 2–25 kDa; Shimadzu: 3–20 kDa). Non-identifiable spectra mostly contained few data counts (n = 10) or low intensity peaks (n = 3), and these spectra could not be identified with the Bruker Biotyper software either. Two spectra of good quality did not have sufficient matches for automated identification with superspectra, though their identification (*Ph. argentipes*, *Ph. perniciosus*) was possible with manual full comparison.

## Conclusions

The power of mass spectrometric identification of organisms depends on the quality of the available database (i.e., reference quality, taxonomic coverage) and its accessibility. The first aspect requires the participation of expert taxonomists, particularly when dealing with closely related species, and experienced users of mass spectrometers who keep a high quality standard for generating the spectra. It further advocates for a centralized, comprehensive database, rather than scattered in-house ones. Accessibility (i.e., the analysis of specimens by third parties) might be an issue at scientific institutions where capacity for analysis, availability and willingness of an operator could be limiting factors. Therefore, a centralized database and analyses at a private company, which provides a customer-oriented service, is a valuable alternative for greater sustainability. As shown in the present work, spectra obtained with different instruments can be analysed using such a centralized database, and this should be possible in the near future via an online platform in a cost-efficient manner.
